# Dynamic planar scintigraphy for the rapid kinetic measurement of myocardial ^123^I-MIBG turnover can identify Lewy body disease

**DOI:** 10.1186/s13550-021-00864-w

**Published:** 2021-12-14

**Authors:** Yoshitaka Kumakura, Yuji Shimizu, Masatsugu Hariu, Ken-ichi Ichikawa, Norihito Yoshida, Masato Suzuki, Satoru Oji, Shinya Narukawa, Haruo Yoshimasu, Kyoichi Nomura

**Affiliations:** 1 Department of Diagnostic Radiology and Nuclear Medicine, Saitama Medical Center (SMC), Saitama Medical University (SMU), 1981 Kamoda, Kawagoe, Saitama 350-8550 Japan; 2 Department of Radiation Oncology, SMC, SMU, Kawagoe, Japan; 3 Radiology Center, SMC, SMU, Kawagoe, Japan; 4 Department of Neurology, SMC, SMU, Kawagoe, Japan; 5 Department of Psychiatry, SMC, SMU, Kawagoe, Japan

**Keywords:** ^123^I-MIBG, Kinetic modelling, Turnover, Lewy body diseases, Myocardial sympathetic nerves

## Abstract

**Background:**

Using two static scans for ^123^I-meta-iodobenzyl-guanidine (^123^I-MIBG) myocardial scintigraphy ignores the dynamic response from vesicular trapping in nerve terminals. Moreover, the long pause between scans is impractical for patients with Lewy body diseases (LBDs). Here, we optimized indices that capture norepinephrine kinetics, tested their diagnostic performance, and determined the differences in ^123^I-MIBG performance among disease groups.

**Methods:**

We developed a new 30-min protocol for ^123^I-MIBG dynamic planar imaging for suspected LBD patients. Pharmacokinetic modelling of time-activity curves (TACs) was used to calculate three new indices: unidirectional uptake of ^123^I-MIBG to vesicular trapping (iUp), rate of myocardial ^123^I-MIBG loss (iLoss), and non-specific fractional distribution of ^123^I-MIBG in the interstitial space. We compared the performance of the new and existing indices with regard to discrimination of patients with or without LBDs. Subgroup analysis was performed to examine differences in ^123^I-MIBG turnover between patients in a dementia with Lewy bodies (DLB) group and two Parkinson’s disease (PD) groups, one with and the other without REM sleep behaviour disorder (RBD).

**Results:**

iLoss was highly discriminative, particularly for patients with low myocardial ^123^I-MIBG trapping, and the new indices outperformed existing ones. ROC analysis revealed that the AUC of iLoss (0.903) was significantly higher than that of early HMR (0.863), while comparable to that of delayed HMR (0.892). The RBD-positive PD group and the DLB group had higher turnover rates than the RBD-negative PD group, indicating a potential association between prognosis and iLoss.

**Conclusion:**

^123^I-MIBG turnover can be quantified in 30 min using a three-parameter model based on ^123^I-MIBG TACs. The discriminatory performance of the new model-based indices might help explain the neurotoxicity or neurodegeneration that occurs in LBD patients.

**Supplementary Information:**

The online version contains supplementary material available at 10.1186/s13550-021-00864-w.

## Introduction

^123^I-meta-iodobenzyl-guanidine (^123^I-MIBG) planar scintigraphy is used to evaluate the noradrenergic integrity of sympathetic nerve terminals in the myocardium. As confirmed by the loss of tyrosine hydroxylase immunoreactivity associated with Lewy body diseases (LBDs) [1], low accumulation of ^123^I-MIBG in the myocardium can serve as a biomarker of LBDs [2–5]. Specifically, semiquantitative indices such as the heart-to-mediastinum ratio (HMR) can discriminate Lewy body diseases with high sensitivity and specificity [6–11]. Unlike the HMR, the myocardial washout rate (WR) is not frequently used in research due to errors caused by low ^123^I-MIBG counts in the affected myocardium [6, 7, 10, 11].

The current protocol for determination of the HMR twice is suboptimal for an accurate LBD diagnostic test. The first scan ignores the first-pass extraction of ^123^I-MIBG, while the second does not account for the constant loss of ^123^I-MIBG from the myocardium. In some circumstances, the second scan might be considered redundant [12]. In addition, the lengthy pause after the first scan could increase patient discomfort. Furthermore, the current protocol does not make full use of ^123^I-MIBG kinetics which can, per se, trace the pathway of norepinephrine in the nerve terminals. Thus, there is ample room for improvement of this two-scan protocol, and novel modifications could address the shortcomings in assessing the kinetics of ^123^I-MIBG accumulation.

Several cardiac PET studies using positron-labelled catecholamine analogues provide good references for the determination of the ^123^I-MIBG scan duration. Tracer equilibrium occurs 30–60 min after the injection of ^11^C-phenylephrine [13], ^11^C-meta-hydroxyephedrine [14, 15], ^18^F-LMI1195 [16], ^18^F-fluoro-hydroxyphenethylguanidines [17, 18] or ^18^F-labelled catecholamines [19–21]. Since these reports were derived from studies in primate species, we infer that long dynamic scans may not be needed for ^123^I-MIBG.

The life cycle of norepinephrine is highly dynamic, and injected ^123^I-MIBG is probably transferred back and forth continuously between intra- and extracellular spaces. However, ^123^I-MIBG cycling cannot be measured via static scintigraphy. The time-activity curves (TACs) of ^18^F-labelled dopamine during uptake and loss are different in denervated terminals and impaired vesicles [22]. Thus, the limitations of static scans of ^123^I-MIBG with regard to assessing nerve viability in patients with advanced LBDs may be overcome by the use of ^123^I-MIBG TACs.

To meet a clinical need for shorter scan protocols, we developed and optimized a novel method of dynamic planar scintigraphy (DPS) for ^123^I-MIBG. To address the shortcoming of the existing ^123^I-MIBG indices, we quantified the rates of uptake and loss of ^123^I-MIBG in the myocardium using DPS for a cohort of consecutively enrolled patients. Since REM sleep behaviour disorder is a strong predictor of cognitive decline and development of dementia in Parkinson’s disease [23, 24], subgroup analysis was also performed. We found that the kinetics of ^123^I-MIBG accumulation could be extracted from a 30-min TAC and that the new kinetic indices had comparable or better discriminatory performance for LBD patients than the existing indices, particularly when used with a machine learning classifier. The resulting improvement in diagnostic performance enhances the clinical value of ^123^I-MIBG scintigraphy as a biomarker of LBDs.

## Materials and methods

### Patient recruitment

This study was approved by the Ethics Committee of the Saitama Medical Center, Saitama Medical University. Eligible patients were evaluated for suspicion of Lewy body disease by neurology and psychiatry specialists. Informed consent was obtained from all participants. Scanning of 250 consecutive participants (mean age: 70.7, 128 men and 122 women) was performed using ^123^I-meta-iodobenzyl guanidine scintigraphy (MyoMIBG, FUJIFILM Toyama Chemical) from October 2017 through April 2019. Additionally, diagnoses of probable rapid-eye movement (REM) sleep behaviour disorder (pRBD) were made based on the responses to the REM Sleep Behaviour Disorder Screening Questionnaire (RBDSQ) [25] and the REM Sleep Behaviour Disorder Single-question Screen Questionnaire (RBD1Q) [26]. After reviewing the patient medical records, those with ischaemic heart disease, congestive heart failure, diabetes, or medications that could affect ^123^I-MIBG imaging were excluded.

### Scanning protocol

^123^I-MIBG scans were performed with a two-detector single-photon emission computerized tomography (SPECT) camera (Discovery NM 630, GE Healthcare) equipped with extended low-energy general-purpose (ELEGP) collimators. ELEGP is optimal for ^123^I imaging due to its high sensitivity and low septal penetration. A 10% energy window was used on the 159-keV photopeak. Planar images were obtained using a 256 × 256 matrix. Scan 1 (30 × 2 s + 40 × 6 s + 75 × 20 s, total 30 min) was started immediately after a bolus injection of 111 MBq of ^123^I-MIBG. Scan 2 (3 × 300 s) and scan 3 (3 × 300 s) were started 90 and 180 min, respectively, after the injection.

### Preparation for plasma input and tissue output functions

To perform kinetic analysis for DPS, we obtained decay-corrected TACs of mediastinal ROIs (mROIs) and heart ROIs (hROIs) using Smart MIBG software [27]. We fitted the mediastinal TACs (mTACs) to a three-phase exponential function with a time offset and a constant term from each peak time through 30, 105, or 195 min after the injection using MATLAB R2018b (MathWorks, Natick, MA, USA). The fitted mTACs were corrected both for ^123^I-MIBG binding to platelets and for metabolites in the plasma. Instead of analysing blood samples with high-performance liquid chromatography (HPLC), we used a population-based blood-to-plasma ratio (BPR) curve and a population-based metabolite correction (PBMC) curve using previously published methods [28]. We set the minimum BPR to 0.6 (haematocrit: 40%) for the first 45 s. Finally, the plasma input functions (PIFs: cps/pixel) of ^123^I-MIBG were obtained by multiplying the fitted mTACs by PBMC/BPR. The tissue TACs (tTACs) of ^123^I-MIBG were obtained by subtracting the fitted mTACs from the heart TACs (hTACs). These PIFs and tTACs were then used for the subsequent analyses.

### Kinetic analysis

In order to describe the kinetics of ^123^I-MIBG, and alongside the conventional ratio indices, we defined three new indices for ^123^I-MIBG DPS, as shown in Table 1: iUp, uptake rate; iLoss, loss rate, and iNs, non-specific distribution. We used a one-tissue three-parameter model (1T3P) defined by the following equation to determine these indices:

1 $${\text{tTAC}}\left( t \right) = {\text{PIF}}\left( t \right) \otimes {\text{iUp}} \cdot \exp \left( { - {\text{iLoss}} \cdot t} \right) + {\text{iNs}} \cdot {\text{PIF}}\left( t \right)$$

where $\otimes$ denotes the convolution operation. A one-tissue two-parameter model (1T2P) was defined by omitting the iNs term from Eq. 1. tTACs of different frame durations (from 1 min to 5, 10, 15, 20, 25, 30, 105, and 195 min) were fitted to both model equations. Weighted nonlinear least-squares optimization was performed with MATLAB functions with a simple weighting of each frame duration. The Akaike information criterion (AIC) [29] and the Schwarz information criterion (SIC) [30] were calculated as follows to compare the model fits:

2A $${\text{AIC}} = N \cdot \ln \left( {{\text{WSSR}}} \right) + 2 \cdot p$$

2B $${\text{SIC}} = N \cdot \ln \left( {{\text{WSSR}}} \right) + p \cdot \ln \left( N \right)$$

where *N* is the number of fitted frames, *p* is the number of parameters, and WSSR is the weighted sum of squared residuals. Then, we used linear and nonlinear regressions to predict HMRs of the early and delayed phases (15 and 195 min, respectively) and WR from the values of iUp and iLoss of the cohort.

**Table 1:** Nomenclature of the kinetic indices for myocardial ^123^I-MIBG scintigraphy

Index	Definition
iUp (min^−1^)	Transfer rate of ^123^I-MIBG from plasma to trapping in the terminals
iLoss (min^−1^)	Loss rate relative to the trapped ^123^I-MIBG
iNs (unitless)	Non-specific distribution of ^123^I-MIBG in the myocardial interstitial fluid
iUp/iLoss	A composite DPS index of the specific distribution of ^123^I-MIBG
eHMR	Heart-to-mediastinum ratio (early: 10–15 min)
dHMR	Heart-to-mediastinum ratio (delayed: 190–195 min)
WR	Washout rate of hROI (mROI counts subtracted and decay-corrected)

^*123*^*I-MIBG* ^123^I-meta-iodobenzyl-guanidine, *DPS* dynamic planar scintigraphy, *hROI* heart ROI, *mROI* mediastinal ROI

### Comparison of diagnostic performance

We sought to compare the classification performance of existing indices between LBD and non-LBD patients with that of our new indices, iUp/iLoss (specific distribution) and iLoss. Patients were considered unclassifiable and thus excluded from this analysis if they had an inconclusive diagnosis, or concurrent LBD and non-LBD. To quantify the diagnostic performance, we used the values of the area under the ROC curve (AUC). To test for significant differences in AUCs between the indices, we used a bootstrap test of the pROC package for R. Then, to apply a machine learning (ML) classifier that takes multiple indices, we used two support vector machines (SVMs) of the scikit-learn package for classification between LBD and non-LBD patients; one SVM employed a linear kernel, and the other, a radial basis function (RBF) kernel [31] in the space of iLoss and iUp/iLoss. The imbalance in the sample size between LBD and non-LBD patients was corrected using the synthetic minority oversampling technique (SMOTE) [32]. The patient cohort was randomly split such that 70% of the patients were used to train the SVMs and the remaining 30% were used to test them. This random splitting was performed 200 times to estimate the diagnostic odds ratios (DORs) as well as AUCs associated with each of the indices.

### ***Comparison of ***^***123***^***I-MIBG turnover among LBD subgroups***

We sorted the patients with Lewy body diseases into three subgroups as follows: Parkinson’s disease (PD) with probable REM sleep behaviour disorder (pRBD), PD without pRBD, and dementia with Lewy bodies (DLB). Differences in the mean values of iUp/iLoss and in the mean values of iLoss were assessed between the PD without pRBD subgroup (as the reference group) and the other two subgroups with Dunnett’s multiple comparison test. We used GraphPad Prism 8 (GraphPad Software, San Diego, CA, USA), R 4.0.2 (R Core Team), and scikit-learn 0.23.2 for Python 3.7.6 (Python Software Foundation) as needed.

## Results

### Patient demographics

After excluding 42 patients who met the exclusion criteria, a total of 208 patients (106 men and 102 women) were included in the kinetic analysis (105 LBD patients, 61 non-LBD patients, and 42 patients with unclassifiable parkinsonism at the final diagnosis). The demographic profiles of the 208 patients, including age, sex, and prevalence of pRBD, are shown in Tables 2 and 3. Table 2 is based on primary diagnoses prior to ^123^I-MIBG, while Table 3 shows final clinical diagnoses.

**Table 2:** Demographics of the 208 patients subjected to pharmacokinetic analysis

	*n*	Age (SD)	Male (%)	pRBD (%)
PD	97	71 (8.5)	46 (47.4)	36 (37.1)
DLB	9	73.3 (7.5)	5 (55.6)	7 (77.8)
PS	86	68.9 (10)	49 (57.0)	11 (12.8)
AD	1	64	0	0
Other	15	72.4 (8.4)	6 (40.0)	3 (20.0)

Demographics by *primary* diagnoses. *PD* Parkinson’s disease, *DLB* dementia with Lewy bodies, *PS* parkinsonian syndrome, unclassified, *AD* Alzheimer’s disease, *pRBD* probable REM sleep behaviour disorder. The age range of the 208 patients was from 43 to 88 (mean ± SD, 70.3 ± 9.1)

**Table 3:** Demographics of the 166 patients subjected to receiver operating characteristic (ROC) curve analysis

	*n*	Age (SD)	Male (%)	pRBD (%)
PD	96	71 (8.5)	45 (46.9)	35 (36.5)
DLB	9	73.3 (7.5)	5 (55.6)	7 (77.8)
MSA	13	68.9 (5.0)	9 (69.2)	2 (15.4)
PSP	11	71.7 (8.6)	7 (63.6)	1 (9.1)
VaP	9	73.4 (6.0)	6 (66.7)	1 (11.1)
DIP	4	69.3 (17.4)	1 (25.0)	0
ET	7	68.0 (12.4)	3 (42.9)	1 (14.3)
CBD	2	61.5 (17.7)	2 (100)	0
Other	15	72.4 (8.4)	6 (40.0)	3 (20.0)
All	166	71.0 (8.6)	84 (50.6)	50 (30.1)

Demographics by confirmed clinical diagnoses. *MSA* multiple system atrophy, *PSP* progressive supranuclear palsy, *VaP* vascular parkinsonism, *DIP* drug-induced parkinsonism, *ET* essential tremor, *CBD* corticobasal degeneration

### Kinetic analysis

**Fig. 1:**
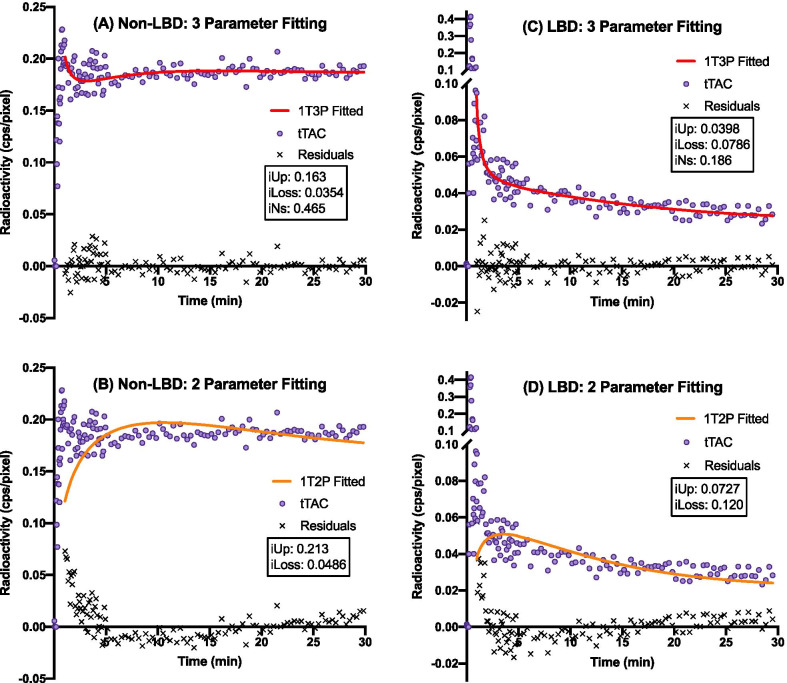
The results of nonlinear least-square fitting of the two kinetic models for two representative patients. **A** and **B** are obtained from a patient without LBD, while **C** and **D** are obtained from a patient with LBD. The tTACs were fitted with three parameters (1T3P: **A**, **C**) better than with two parameters (1T2P: **B**, **D**). See Additional file 1: Figs. 1 and 2 for the corresponding ROI TACs and PIFs

We first generated TACs from ROIs drawn on patient images (Additional file 1: Fig. 1). After extracting the PIFs and tTACs (Additional file 1: Fig. 2), we proceeded to fit these curves to two models with and without the third index (iNs). As shown for two representative non-LBD and LBD patients in Fig. 1, the 1T3P model provided better fitting than the 1T2P model. The kinetic results are summarized in Table 4 for 1T3P and in Table 5 for 1T2P. The information criteria (AIC and SIC) were lower with 1T3P than with 1T2P. In the 1T3P series, the lowest values (AIC: − 558.5; SIC: − 550.3) were obtained at 30 min. Truncating the TACs to less than 30 min led to negative values of iUp and iLoss, while prolonging TAC acquisition to 105 or 195 min provided no additional benefit. Using the same cohort, the mean (SD) values of early HMR, delayed HMR and WR were 1.98 (0.52), 1.98 (0.77), and 0.38 (0.30), respectively. Thus, using the data from tTACs spanning 1–30 min in the 1T3P model best describes the kinetics of ^123^I-MIBG DPS.

**Table 4:** Kinetic results of the three-parameter model (1T3P)

1T3P	5 min	10 min	15 min	20 min	25 min	30 min	105 min	195 min
iUp	0.159	0.150	0.141	0.135	0.132	0.130	0.140	0.149
	(0.092)	(0.077)	(0.070)	(0.066)	(0.064)	(0.063)	0.061	(0.057)
iLoss	0.058	0.067	0.059	0.054	0.051	0.050	0.061	0.071
	(0.121)	(0.032)	(0.020)	(0.018)	(0.017)	(0.017)	(0.027)	(0.038)
iNs	0.107	0.132	0.162	0.186	0.200	0.209	0.155	0.117
	(0.133)	(0.112)	(0.108)	(0.110)	(0.112)	(0.113)	(0.129)	(0.165)
iUp/iLoss	1.92	2.76	2.65	2.93	3.05	3.10	3.01	3.00
	(2.65)	(4.52)	(1.85)	(1.84)	(1.93)	(1.99)	(2.01)	(2.03)
0.693/iLoss	7.3	16.6	10.5	14.1	14.8	15.2	13.5	12.8
	(18.2)	(77.9)	(23.8)	(4.2)	(4.3)	(4.4)	(5.5)	(6.3)
Akaike IC	− 203.7	− 287.9	− 360.5	− 428.4	− 494.1	− 558.5	− 551.1	− 551.9
Schwarz IC	− 198.6	− 281.9	− 353.8	− 421.0	− 486.3	− 550.3	− 542.8	− 543.5
iUp NegCnt	0	0	0	0	0	0	0	0
iLoss NegCnt	29	5	2	0	0	0	0	0
iNs NegCnt	42	25	11	8	7	3	16	51

Means (and SDs) of the kinetic indices of 1T3P, Akaike’s information criterion and Schwarz’s information criterion. NegCnt: negative counts that were unfavourable; 0.693/iLoss is the pharmacological half-life

**Table 5:** Kinetic results of the two-parameter model (1T2P)

1T2P	5 min	10 min	15 min	20 min	25 min	30 min	105 min	195 min
iUp	0.197	0.179	0.168	0.161	0.156	0.153	0.154	0.158
	(0.088)	(0.080)	(0.076)	(0.073)	(0.071)	(0.069)	(0.066)	(0.064)
iLoss	0.152	0.099	0.080	0.069	0.063	0.060	0.065	0.072
	(0.115)	(0.040)	(0.029)	(0.025)	(0.023)	(0.022)	(0.030)	(0.038)
iUp/iLoss	1.72	2.13	2.49	2.77	2.96	3.08	3.12	3.10
	(1.46)	(1.24)	(1.48)	(1.68)	(1.81)	(1.90)	(2.07)	(2.13)
0.693/iLoss	5.5	7.9	9.7	11.2	12.2	12.9	12.9	12.4
	(12.8)	(2.7)	(3.0)	(3.5)	(3.8)	(4.1)	(5.2)	(5.9)
Akaike IC	− 198.9	− 273.1	− 333.7	− 389.3	− 444.1	− 499.4	− 514.1	− 522.4
Schwarz IC	− 195.5	− 269.1	− 329.2	− 384.4	− 438.9	− 493.9	− 508.6	− 516.8
iUp NegCnt	0	0	0	0	0	0	0	0
iLoss NegCnt	3	0	0	0	0	0	0	0

Means (and SDs) of kinetic indices of 1T2P, Akaike’s information criterion and Schwarz’s information criterion. NegCnt: negative counts that were unfavourable; 0.693/iLoss is the pharmacological half life

**Fig. 2:**
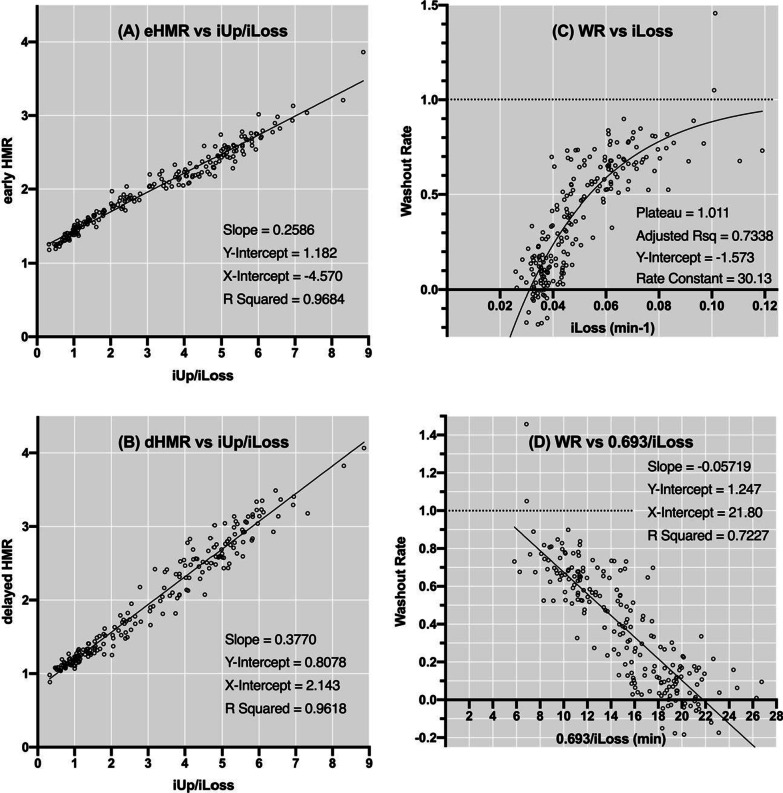
The results of regressions between the existing indices and new indices of ^123^I-MIBG myocardial scintigraphy. Linear regressions between iUp/iLoss and the early (eHMR) (**A**) and delayed HMR (dHMR) (**B**) are shown. A nonlinear regression with an exponential monomolecular growth model between iLoss and WR (**C**) can be converted to a linear regression between 0.693/iLoss and WR (**D**)

**Fig. 3:**
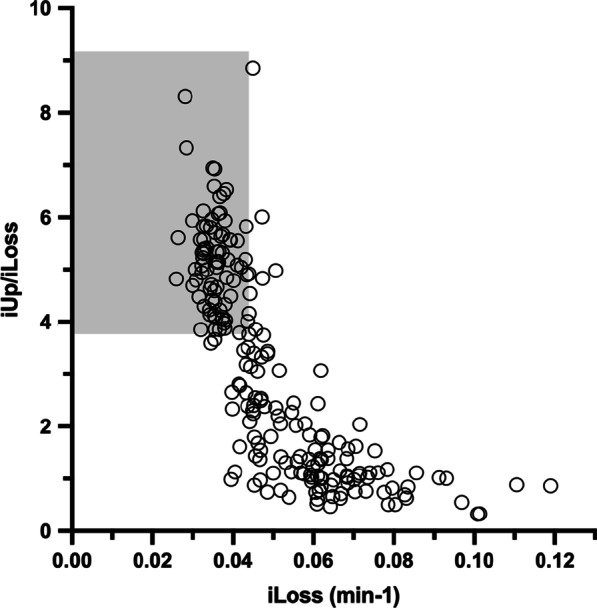
The scatter plot of iLoss (min^−1^) versus iUp/iLoss obtained from 208 patients. The shaded rectangle represents a territory based on the cut-off values for iLoss and iUp/iLoss shown in Table 6. Note that high values of iLoss were counterbalanced by iUp to some extent

We next sought to construct predictors of the existing indices to ensure follow-up and continuity. Linear regression analysis revealed that iUp/iLoss was an excellent predictor of early HMR (Fig. 2A) and delayed HMR (Fig. 2B). The scatter plot between iLoss and WR indicated a good fit with an exponential monomolecular growth model (Fig. 2C). The pharmacological half-life of trapped ^123^I-MIBG (0.693/iLoss) was a good linear predictor of WR (Fig. 2D). The scatter plot of iLoss and iUp/iLoss for the 208 patients and the cut-off values for each parameter are shown in Fig. 3.

### ***Superior diagnostic performance of ***^***123***^***I-MIBG DPS***

**Fig. 4:**
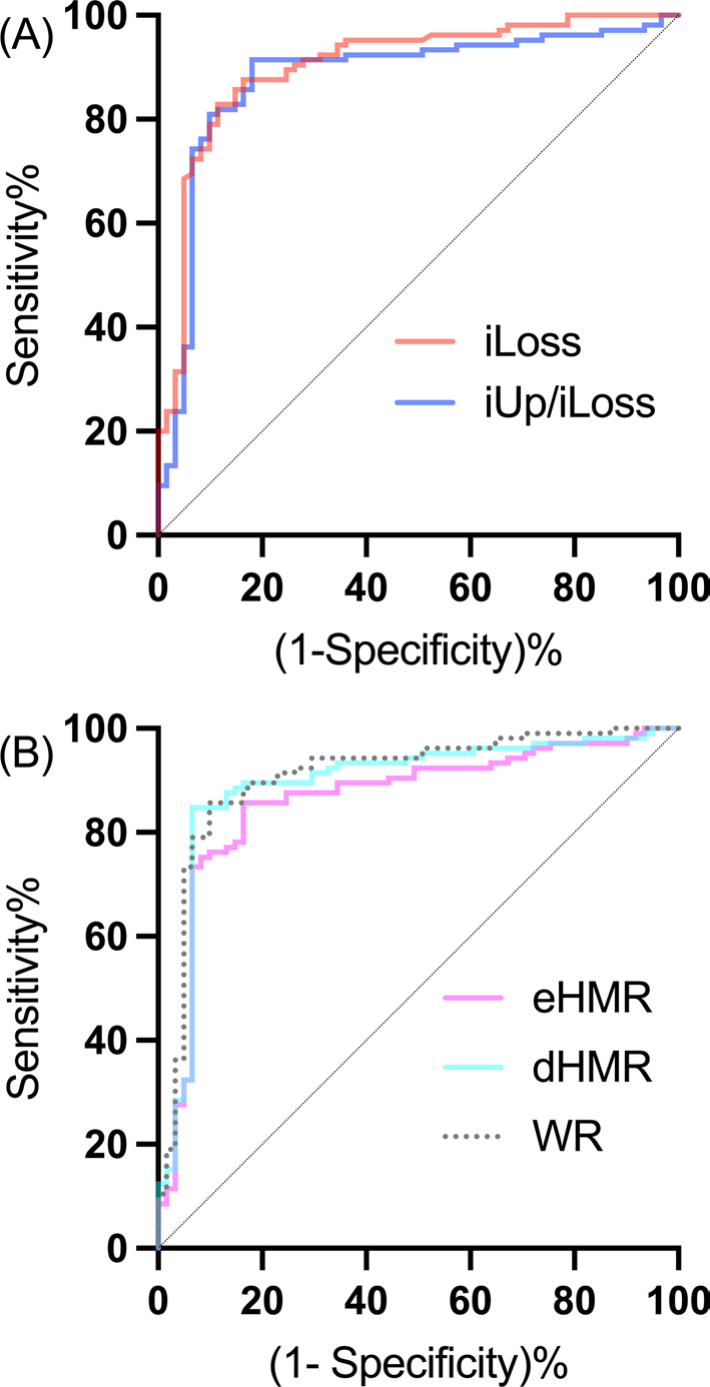
Receiver operating characteristic (ROC) curves of the new (**A**) and current (**B**) indices of ^123^I-MIBG for identifying patients with Lewy body disease (LBD). ROC analysis was performed for 166 tests (see the demographics of the 105 LBD and 61 non-LBD patients in Table 3). Areas under the curve (AUCs) are summarized in Table 6

**Fig. 5:**
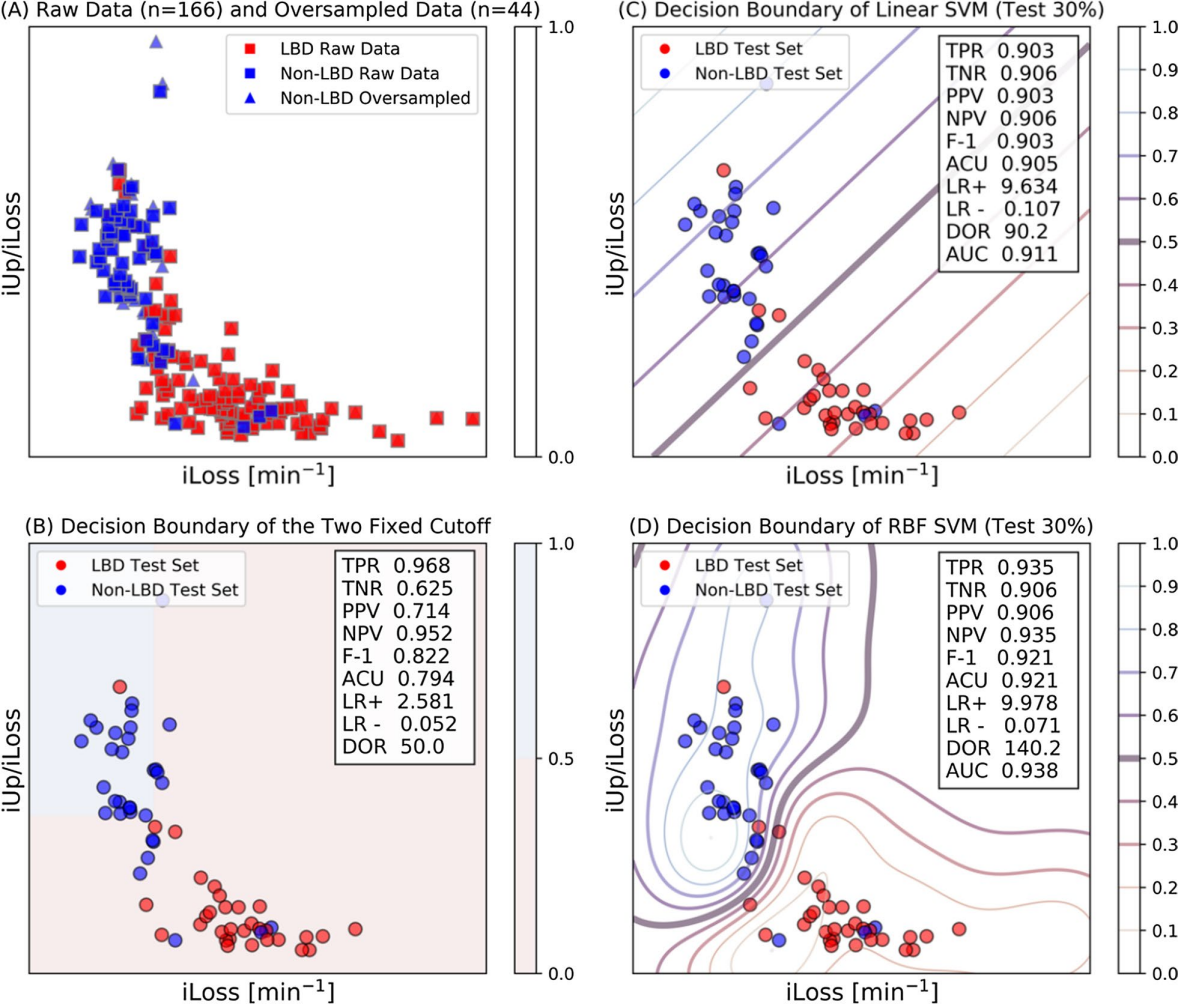
Representative results of machine learning classifier models for iLoss and iUp/iLoss to discriminate patients with Lewy body disease (LBD) from those with non-LBD. After correction for class imbalance using SMOTE, 210 coordinates of iUp and iUp/iLoss, which represent 105 LBD patients (squares in red) and 105 oversampled non-LBD patients (squares and triangles in blue), are shown (**A**). An example portion allocated for testing (63 samples, 30%) is shown with the independent cut-off values of iLoss and iUp/iLoss (**B**). Decision boundaries (thick lines) and predicted probabilities (thin lines) were generated from single runs of the SVM classifier models with the linear kernel (**C**) and the radial basis function kernel (**D**)

To compare the diagnostic performance, 42 patients with unclassifiable parkinsonism were excluded from the ROC analysis. The demographic characteristics of the remaining 166 patients included in this analysis (105 patients with LBDs and 61 non-LBD patients) are shown in Table 2. The results of the ROC analysis are summarized in Table 6. (The corresponding curves are shown in Fig. 4.) The best diagnostic performance was obtained by iLoss, followed by iUp/iLoss. Within short scan protocols, the AUCs of these two indices were significantly higher than that of early HMR. Using iLoss and iUp/iLoss together, representative classification results obtained with single runs of the two SVMs are shown in Fig. 5. After 200 runs, the linear SVM and RBF-SVM gave mean AUCs of 0.911 and 0.916, respectively. The mean DORs were 31.6 for the two fixed cut-off values of iLoss and iUp/iLoss, 56.4 for the linear SVM, and 57.5 for the RBF-SVM, indicating that the SVMs for the two new indices may be able to better discriminate patients with LBDs from those without.

**Table 6:** Results of the receiver operating characteristic (ROC) curve analysis

	AUC	95% CI	Cut-off	Sensitivity	95% CI	Specificity	95% CI
iUp/iLoss	0.8784*	0.8176–0.9391	3.769	0.9143	0.8451–0.9543	0.8197	0.7053–0.8962
iLoss	0.9029*	0.8521–0.9537	0.0436	0.8762	0.7996–0.9262	0.8361	0.7239–0.9084
eHMR	0.8631	0.8011–0.9251	2.042	0.8571	0.7776–0.9115	0.8361	0.7239–0.9084
(dHMR)	0.8924	0.8349–0.9500	1.720	0.8476	0.7667–0.9040	0.9344	0.8432–0.9742
(WR)	0.9085	0.8572–0.9598	0.3485	0.8571	0.7776–0.9115	0.9016	0.8016–0.9541

A total of 166 patients were included. Indices enclosed in parentheses cannot be obtained in 30 min. Asterisks denote significant differences (*: *P* < 0.05) detected by the bootstrap test versus the AUC of eHMR. The cut-off values are those that yield the largest Youden’s index

### ***Comparison of ***^***123***^***I-MIBG turnover among LBD subgroups***

**Fig. 6:**
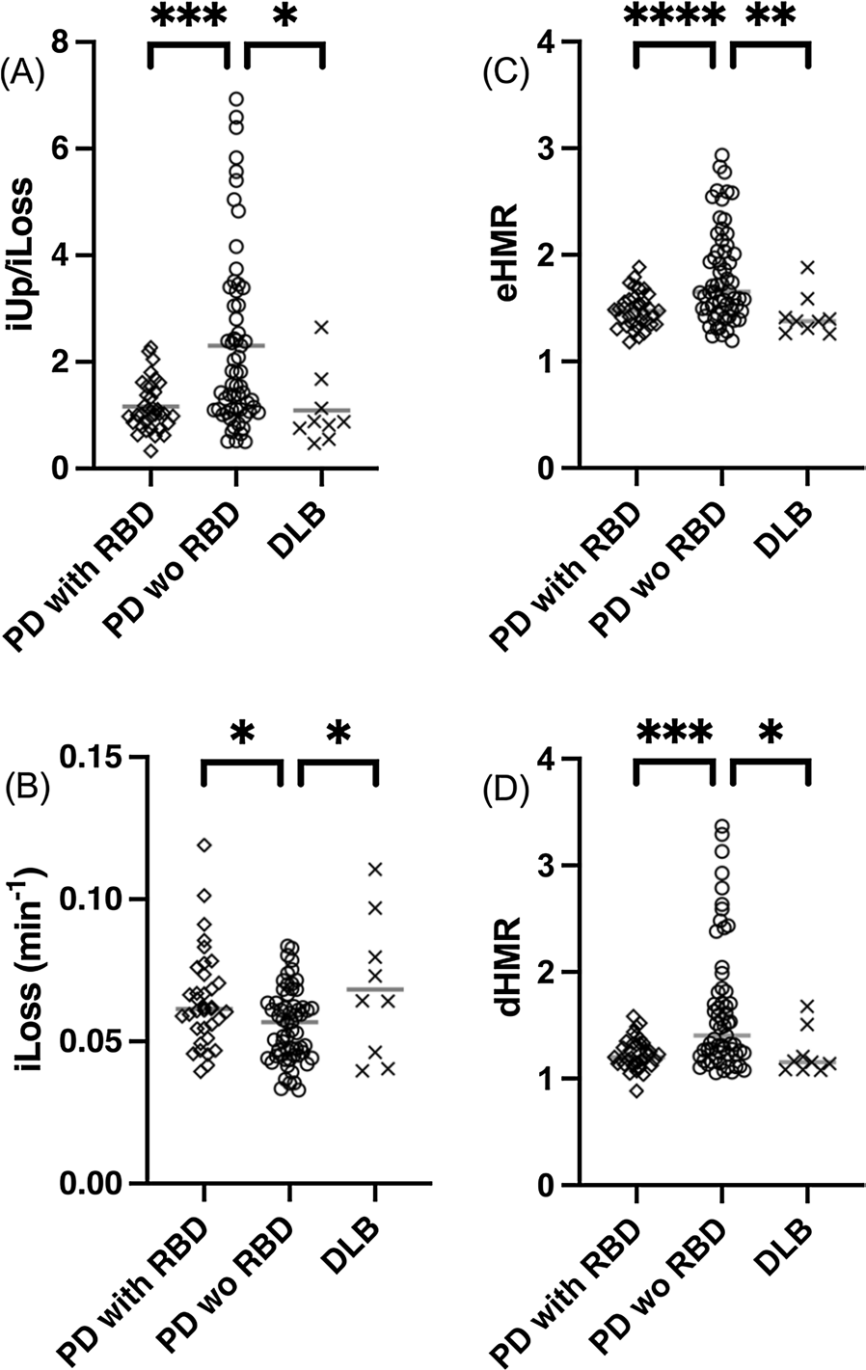
Column scatter plots of iUp/iLoss (**A**), iLoss (**B**), early HMR (**C**) and delayed HMR (**D**) comparing the three subgroups of patients with LBDs. Asterisks denote significant differences (*: *P* < 0.05, **: *P* < 0.01, ***: *P* < 0.001, ****: *P* < 0.0001) detected by Dunnett’s multiple comparison test versus the baseline group (PD without pRBD). Horizontal bars in grey denote the mean values of the three subgroups. Note that some overestimated eHMRs (underestimated severity) in patients with RBD-negative early PD might exaggerate statistical differences

Ninety-six patients had clinically established PD. Among them, 36% (35/96) were pRBD-positive, while 64% (61/96) were pRBD-negative. Nine patients were diagnosed with DLB (Table 3). The mean (SD) estimates of iUp/iLoss were 1.17 (0.47) for pRBD-positive PD patients, 2.30 (1.67) for pRBD-negative PD patients, and 1.09 (0.68) for patients with DLB. Dunnett’s multiple comparison test revealed that the mean iUp/iLoss value of the pRBD-negative PD subgroup was significantly higher than each of the other two subgroups (*P* < 0.001 and 0.05; Fig. 6A). The mean (SD) estimates of iLoss were 0.0647 (0.0170) for pRBD-positive PD, 0.0557 (0.0129) for pRBD-negative PD, and 0.0683 (0.0247) for DLB. Likewise, the mean iLoss value of the pRBD-negative PD subgroup was significantly lower than each of the other two (*P* < 0.05; Fig. 6B). Thus, the diagnostic performances of iLoss and iUp/iLoss can potentially distinguish LBD subgroups. The mean (SD) estimates of early and delayed HMRs were 1.48 (0.16) and 1.24 (0.15) for pRBD-positive PD patients, 1.80 (0.45) and 1.64 (0.60) for pRBD-negative PD patients, and 1.43 (0.20) and 1.23 (0.21) for patients with DLB. The statistical results for HMRs were similar as shown in Fig. 6C, D.

## Discussion

In this study, we sought to optimize the diagnostic procedure for detection of LBDs. ^123^I-MIBG TACs obtained from dynamic imaging for 30 min yielded iLoss and iUp/iLoss values that could be used to distinguish LBD and non-LBD patients with an accuracy equal to or greater than the current standard indices. Thus, iLoss could serve as a biomarker for neurodegeneration in the sympathetic nerve terminals of patients with LBDs.

We used population-based correction functions to obtain plasma input functions of ^123^I-MIBG. Since the plasma ^123^I-MIBG fraction gradually declines to 30% as previously reported [28], simple rescaling of mediastinum TACs does not suffice. The metabolite correction curves cannot be ignored when estimating the kinetic indices, as with the case of ^18^F-FDOPA kinetic modelling [33, 34]. The normal range of iUp/iLoss would be wider than those of HMRs, due to the presence or absence of plasma radioactivity correction for metabolites that do not penetrate the terminals.

In the 1T3P model, we set the interstitial distribution (iNs) of ^123^I-MIBG as a fraction of the PIF. We assumed that equilibration with ^123^I-MIBG in the plasma occurred within 1 min post-injection, in line with the reported equilibration time of the myocardial intensity of Gd-DTPA, an MRI extracellular contrast agent [35]. The 1T3P model stems from an ^18^F-FDOPA model for brain PET that quantifies the turnover according to the same principle [33]. The loss rate of ^123^I-MIBG in planar imaging appears comparable to that of 4D imaging; indeed, the range of iLoss was in good agreement with the normal loss rate (< 0.035) of ^18^F-fluorodopamine [36]. We confirmed that a 30-min scan was sufficiently long compared with the mean of 0.693/iLoss (15.2 min). Presumably, steady-state ^123^I-MIBG trapping was achieved in 30 min by the combination of the bolus-like delivery to the myocardium, the slow plasma radioactivity excretion (Additional file 1: Fig. 1) and the stable ^123^I-MIBG fraction in plasma [28]. These characteristics are common among other radiolabelled catecholamine analogues [13–21].

iUp/iLoss was an excellent predictor of early and delayed HMRs (Fig. 2A, B). However, iUp/iLoss is not simply a presentation of the current standard indices on a different scale; rather, it is an independent indicator of the true trapping of ^123^I-MIBG [34]. Indeed, the y-intercept (1.18) seen in Fig. 2A was approximately the mean iNs (0.21) plus the blood pool factor. WR could be predicted both from iLoss (Fig. 2C) and from the half-life of ^123^I-MIBG (0.693/iLoss) (Fig. 2D), but with limited precision. These predictions for WR are based on crude assumptions, such as ignoring ^123^I-MIBG metabolites (see Appendix). Thus, iLoss stands as a unique index for ^123^I-MIBG.

Our findings revealed that the iLoss and iUp/iLoss values derived from patients with LBDs were spread over a wide range (Figs. 3, 5), replicating a scatter plot of ^18^F-fluorodopamine PET [36]. In essence, deviation from the normal range could be attributed to an increase in iLoss and/or decrease in iUp. Notably, the high loss of ^123^I-MIBG was compensated in part by viable ^123^I-MIBG uptake. Thus, the apparent viability even with extremely low levels of ^123^I-MIBG could represent “sick but not dead” nerve terminals [37]. Unlike iUp, the fractional loss rate is (by definition) independent of ^123^I-MIBG trapping and does not change with the density of intact terminals. Thus, the observed increased loss rate is consistent with the level of hazard to viable terminals.

Alpha-synuclein oligomers are considered crucial in the pathogenesis of LBDs [38], as they impair the homeostasis of synaptic vesicles and membranes [39–41]. The leakage of catecholamines from vesicles causes the accumulation of excess toxic aldehydes in the cytosol. Ultimately, this toxicity causes the aggregation of alpha-synuclein protein. Indeed, the aldehyde metabolite from norepinephrine exacerbates nerve degeneration [42]. Although we were unable to calculate the level of ^123^I-MIBG in the cytosol, the loss rate of ^123^I-MIBG could be used as a surrogate. We presume that the excess extravesicular aldehydes are key to establishing ^123^I-MIBG as a biomarker of LBDs. In particular, the high loss rate of ^123^I-MIBG might be a symptom of the ‘vicious cycle’ that underlies the catecholaldehyde hypothesis [37, 43]. Thus, individual variability in the progression rate of LBDs might be reflected by iLoss of ^123^I-MIBG DPS. Unlike a high iLoss value, a low iUp/iLoss value cannot be used to discriminate between a loss of terminals due to neuron death versus the reduced function of viable nerve terminals due to alpha-synuclein oligomerization.

ROC analysis (Table 6) confirmed the good diagnostic accuracy of the early and delayed HMRs in discriminating LBDs described in previous studies [6–11]. The AUCs of both iLoss and iUp/iLoss were significantly higher than that of early HMR (*P* < 0.05). The highest AUC (0.903) was obtained by the value of iLoss obtained at 30 min. Furthermore, the performance of the new indices obtained by 30-min DPS was on par with that of the current indices obtained at three hours (Table 6, Fig. 4). The additional use of machine learning (ML) is attractive in that oblique or curved cut-off lines can be fixed in the coordinate space. Indeed, our results (Fig. 5) suggest that a ML classifier for multiple indices could outperform a single cut-off point for iLoss.

^123^I-MIBG turnover was higher in the pRBD-positive PD subgroup and the DLB subgroup than in the baseline PD subgroup as indicated by iUp/iLoss and iLoss (Fig. 6). Based on the proportionality between iUp/iLoss and HMRs, these results agreed well with earlier studies using HMRs and similar populations [3, 44–47]. Patients with pRBD-positive PD and DLB tended to have higher iLoss values, which merits further analysis. Patients with DLB have a poorer survival rate than those with PD [48], and their survival rate is influenced by frequent orthostatic hypotension [49]. However, RBD predicts motor progression in patients with PD [50]. Thus, further research is required to explore the prognostic value of ^123^I-MIBG iLoss with regard to distinguishing these LBD subgroups.

In addition to the cyclical nature of some diagnoses, this study has a limitation in that some cases were diagnosed with PD based on the currently used ^123^I-MIBG scintigraphy method. Thus, the ROC curves might be biased in favour of existing indices. We recommend that the new DPS indices be compared with delayed HMRs in future replication studies. Moreover, we had no choice but to omit inconclusive patients due to the nature of our study. Fewer difficult/borderline cases are likely to be included in the diagnostic performance analysis. Thus, the performance of all indices might be systematically overestimated. Although not tested here, we suggest that this method could be applied to diagnosis of congestive heart failure, catecholamine-induced cardiomyopathy (e.g. pheochromocytoma), and presumably treatment-related complications of anti-tumour agents.

## Conclusion

We show that dynamic planar scintigraphy followed by kinetic modelling is an optimal method for using ^123^I-MIBG as a biomarker of LBDs, as it allows a short scan duration of 30 min and quantification of ^123^I-MIBG turnover in the sympathetic nerve terminals. The DPS method reduces the waiting times for patients and their family members before and after the scan while maximizing diagnostic performance. High ^123^I-MIBG turnover might be present in the nerve terminals of patients affected by LBDs that are still viable. If so, the high loss rate of ^123^I-MIBG might be a biomarker of the neurotoxicity caused by LBDs. Further studies are needed to confirm this hypothesis.

## Supplementary Information

**Additional file 1. Supplementary Figure 1**. The mTACs and hTACs obtained from the two representative patients from Figure 1.** Supplementary Figure 2**. The plasma input functions (PIFs) and tissue TACs (tTACs) obtained from the two representative patients from Figure 1.** APPENDIX**.

## Data Availability

Not available due to ethical restrictions.

## Code availability

Available upon reasonable request.
