# Supplementary material for: Dynamic planar scintigraphy for the rapid kinetic measurement of myocardial 123I-MIBG turnover can identify Lewy body disease
Source: EJNMMI Res. 2021 Dec 14;11:122. doi: 10.1186/s13550-021-00864-w (PMC8671580; doi:10.1186/s13550-021-00864-w)
Supplement: Supplementary file 1 — Additional file 1. Supplementary Figure 1. The mTACs and hTACs obtained from the two representative patients from Figure 1. Supplementary Figure 2. The plasma input functions (PIFs) and tissue TACs (tTACs) obtained from the two representative patients from Figure 1. APPENDIX. [file 13550_2021_864_MOESM1_ESM.docx]

**Supplementary Figure 1** The mTACs and hTACs obtained from the two representative patients from Figure 1. The TACs of scan 1 are shown from 0 to 2 min (A, B) and 30 min (C, D), together with the TACs of scan 3. Myocardial accumulation of ^123^I-MIBG was normal for a non-LBD patient (A, C: early HMR: 2.31) and low for an LBD patient (B, D: early HMR: 1.24). The initial trapping was seen immediately after the first pass for the non-LBD patient, while it was minimal for the LBD patient. Three-exponential fits for the mTACs were calculated for denoising (red line). The residuals were distributed normally along the x-axis, indicating good fits.

**Supplementary Figure 2** The plasma input functions (PIFs) and tissue TACs (tTACs) obtained from the two representative patients from Figure 1. The corrections for ^123^I-MIBG platelet binding and metabolites resulted in larger peaks in the PIFs than those in the mTACs (Supplementary Figure 1). The PIFs appeared to equilibrate with the tTACs within 30 min. Note that the tTACs stabilize earlier than the PIFs.

**APPENDIX**

The exponential monomolecular growth model used for the nonlinear regression between iLoss and WR in Figure 2C assumes that the impulse function represents the bolus delivery of ^123^I-MIBG to the myocardium and that the tissue TACs approximate a single exponential decay. If the amount of trapped ^123^I-MIBG at time *T* can be represented as exp(-*iLoss*⋅*T*) multiplied by the amount at time zero (*A*_0_), the washout rate would be:

*WR* = 1 – exp(*Td* -*Te*)⋅*iLoss*

where *Te* and *Td* are the times at the midpoints of the early and delayed scans.

However, this simple model does not take into account the contamination of the myocardium with ^123^I-MIBG metabolites. Thus, the measured values of iLoss can predict those of WR with limited accuracy, and vice versa.
